# The Next Revolution: Percutaneous Aortic Valve Replacement

**DOI:** 10.5041/RMMJ.10016

**Published:** 2010-10-31

**Authors:** Martin B. Leon, Eugenia Nikolsky

**Affiliations:** 1 Professor of Medicine, Columbia University College of Physicians and Surgeons, Associate Director, Center for Interventional Vascular Therapy, New York-Presbyterian Hospital/Columbia University Medical Center and Cardiovascular Research Foundation, New York, NY, USA;; 2 Senior Lecturer, Technion – Israel Institute of Technology, and Director, Cardiovascular Research Unit and Intermediate Cardiac Care Unit, Rambam Health Care Campus, Haifa, Israel

**Keywords:** Aortic valve, percutaneous, transcatheter

## Abstract

Aortic valve replacement (AVR) is a treatment of choice for patients with symptomatic severe aortic stenosis (AS). However, a significant proportion of these patients do not undergo surgical AVR due to high-risk features. Transcatheter aortic valve implantation (TAVI) has emerged as an alternative for patients with severe AS who are not candidates for open-heart surgery. Since the introduction of TAVI to the medical community in 2002, there has been an explosive growth in procedures. The balloon-expandable Edwards SAPIEN valve and the self-expanding CoreValve ReValving^TM^ system contribute the largest patient experience with more than 10,000 patients treated with TAVI to date. Clinical outcomes have stabilized in experienced hands, with 30-day mortality less than 10%. Careful patient selection, growing operator experience, and an integrated multidisciplinary team approach contribute to notable improvement in outcomes. In the first randomized pivotal PARTNER trial, in patients with severe AS not suitable candidates for surgical AVR, TAVI compared with standard therapy, significantly improved survival and cardiac symptoms, but was associated with higher incidence of major strokes and major vascular events. The results of randomized comparison of TAVI with AVR among high-risk patients with AS for whom surgery is a viable option are eagerly awaited to provide further evidence on the applicability of TAVI in these patients.

Transcatheter heart valve therapy has emerged as an alternative for patients with valvular heart disease who are not candidates for open-heart surgery. In 2000, Philipp Bonhoeffer, from Great Ormond Street Hospital, London, UK, successfully performed the world’s first percutaneous pulmonary valve implantation,^1^ and 2 years later Alain G. Cribier, from Hôpital Charles Nicolle, Rouen, France, carried out the first-in-man percutaneous aortic valve implantation.^2^ Since then transcatheter technology has evolved tremendously and has now the potential to transform the management of several common heart conditions.

## AORTIC STENOSIS: THE SCOPE OF THE PROBLEM

**Figure 1:**
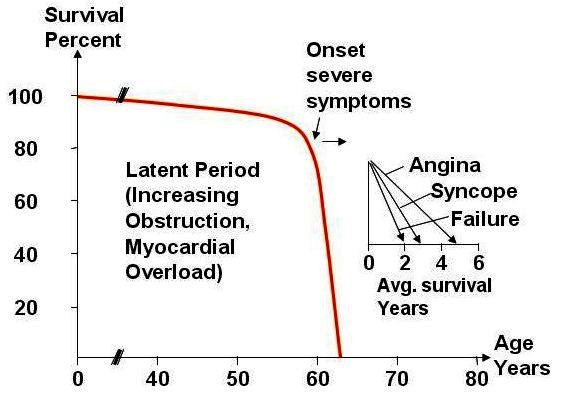
Survival after onset of symptoms in patients with severe aortic stenosis. Figure included with permission from Circulation (Ross & Braunwald. Circulation 1968;38:61)^4^

Demographic status in the developed nations is facing a transition in which the proportion of the population over the age of 65 years will double in the next few decades.^3^ Due to increased life expectancy for this age group more than a quarter of the population older than 65 years of age are expected to live until age 90. Aging of the population has resulted in the escalating prevalence of severe aortic stenosis (AS), the most common form of valvular heart disease in adults. Until relatively late in the course of the disease the patients are free from cardiovascular symptoms. However, once the symptoms manifest, the prognosis is poor: the onset of angina, syncope, and/or dyspnea has been shown to correlate with an average time to death of 5, 3, and 2 years, respectively (Figure 1).^4^,^5^ Surgical aortic valve replacement (AVR) is the treatment of choice and should be performed promptly once even minor symptoms occur.^6^ The operation results in the improvement of survival, symptoms, hemodynamic parameters, left ventricle systolic function, as well as in remodeling and reverse of left ventricular mass.^7^–^9^

Still, many patients, particularly the elderly with major co-morbidities, do not undergo potentially beneficial interventions for severe AS.^10^,^11^ Specifically, analysis of the therapeutic decisions in the Euro Heart Survey on valvular heart disease including 5,001 patients from 92 centers in 25 European countries in 2001 showed that 32% of symptomatic patients with severe AS were denied surgery.^10^ The main reasons for the absence of surgical intervention are high-risk features of the population including old age, presence of serious co-morbidities, and impaired left ventricular function.^10^ Importantly, more than half of the patients with severe AS patients are older than 75 years of age, and by estimation, in the USA, the potential AS treatment cohort could exceed 250,000 patients.^11^ Thus, there is an unmet clinical need for transcatheter techniques that offer an alternative to surgical AVR and could significantly reduce morbidity and mortality of patients with severe AS who are not currently considered for surgery.

## CURRENT PERCUTANEOUS AORTIC VALVE MODELS

**Figure 2:**
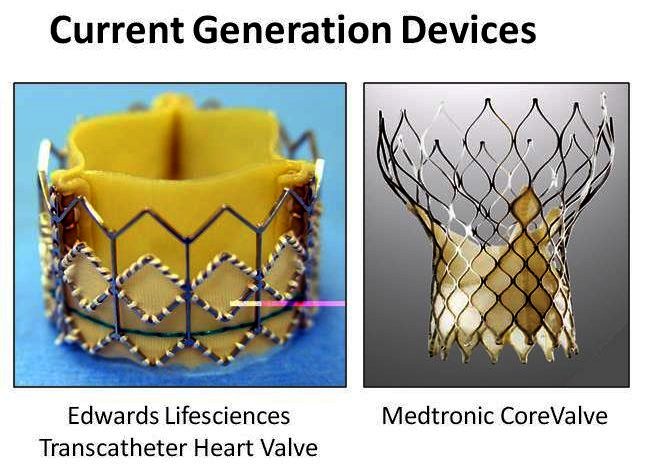
The balloon-expandable Edwards SAPIEN valve (Edwards Lifescience, Irvine, California) and the self-expanding CoreValve ReValving^™^ System (Core-Valve Inc., Irvine, California).

Several aortic valve prototypes have been reported and are currently in different stages of development. The balloon-expandable Edwards SAPIEN valve (Edwards Lifescience, Irvine, California, USA) and the self-expanding CoreValve ReValving^™^ system (CoreValve Inc., Irvine, California) contribute the largest patient experience with more than 10,000 patients treated with transcatheter aortic valve implantation (TAVI) to date (Figure 2).

**Figure 3:**
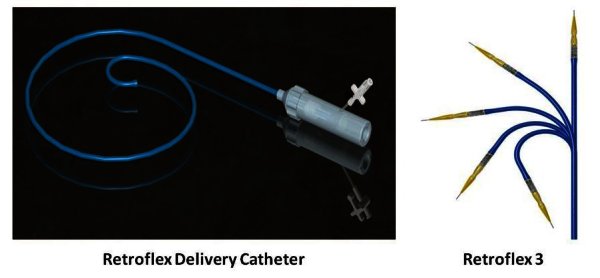
Edwards Flex Catheter Delivery System.

The valves’ design is in the process of constant refinement to gain optimal device delivery, performance, and durability. The earliest version of Cribier-Edwards’ transcatheter heart valve (THV) existed only in the size of 23 mm, the second-generation Edwards SAPIEN valve became available in size 26 mm, while the third-generation, SAPIEN THV, will also exist in sizes of 20 and 29 mm to achieve optimal matching with the aortic annulus. The leaflet geometry has been improved as well, allowing better performance and longer durability of the device. The latest version of the THV consists of three bovine scallop-shaped pericardial leaflets mounted within a tubular slotted stainless steel balloon-expandable stent. The delivery system for the transcatheter valve is a critical component in facilitating the delivery and implantation of the valve through femoral approach. The last, third, generation of the RetroFlex delivery system (Figure 3) is advanced through a hydrophilic 18-Fr sheath (compared to 22-Fr and 24-Fr sheaths in the earlier versions). This delivery system optimizes the ability to control the navigation of the valve, through the incorporation of the valve expansion balloon directly into the delivery system’s flexible tip, and facilitates the crossing of the patient’s native aortic valve.

Implantation of the Edwards valve may be performed using anterograde (transseptal), retrograde (transfemoral or subclavian), or apical approach. Anterograde technique was used in the early stages of procedure development. It was challenging due to the necessity for a transseptal puncture and the passage of the catheter/valve across the mitral valve which usually created a poorly tolerated acute mitral regurgitation. For patients without adequate femoral access (e.g. excessive tortuosity, heavy calcification, or insufficient minimal luminal diameter of the aorta, iliac, and/or femoral arteries to accommodate the delivery catheter) the transapical approach was developed to avoid vascular and embolic complications. A minimal left thoracotomy allows exposure of the left ventricle apex. A soft guide-wire is passed in an antegrade fashion across the stenotic aortic valve under fluoroscopic and echocardiographic guidance. A sheath is introduced and positioned across the aortic valve. After performing balloon valvuloplasty, a transapical delivery sheath is inserted. The valve is then delivered and implanted using the special application system.

**Figure 4:**
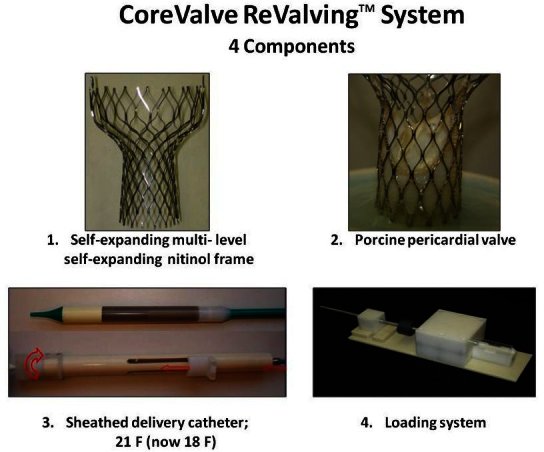
Four components of CoreValve ReValving^™^ System.

The CoreValve ReValving^™^ system is a self-centering and partially repositionable one, allowing for more liberty during deployment. The system has four main components, including self-expanding multilevel nitinol frame, porcine pericardial valve, sheathed delivery catheter (previously 21-Fr and 24-Fr, and now 18-Fr), and loading system (Figure 4). The valve implantation is usually performed through the femoral approach. The valve is mounted in a self-expanding nitinol frame that extends from the left ventricular outflow tract into the aortic root. The frame has three dedicated functional areas that allow proper orientation, anchoring, and valve placement. The valve rests in a constrained supra-annular position avoiding interference with the coronary ostia. The prosthesis exists in two sizes of 26 mm and 29 mm to fit into an aortic annulus of 20–23 mm or 24–27 mm, respectively. In patients in whom femoral access is not feasible, the valve implantation may be successfully performed using the subclavian or axillar arterial approach.^12^ Two cases of successful “off-pump” transaortic implantation of the prosthetic aortic valve via the direct puncture of the ascending aorta, accessed through a mini-sternotomy, have been also described.^13^

Accurate positioning of the implant, selecting the optimal projection for valve implantation, selecting the valve size, and evaluating the post-procedure results immediately after valve deployment may be facilitated by a novel real-time imaging modality capable of three-dimensional reconstruction of the ascending aorta (CardioOp-THV, C-THV, Paieon Inc., Park Afek, Israel).^14^

## CLINICAL TRIAL RESULTS

### DATA SOURCES

**Figure 5:**
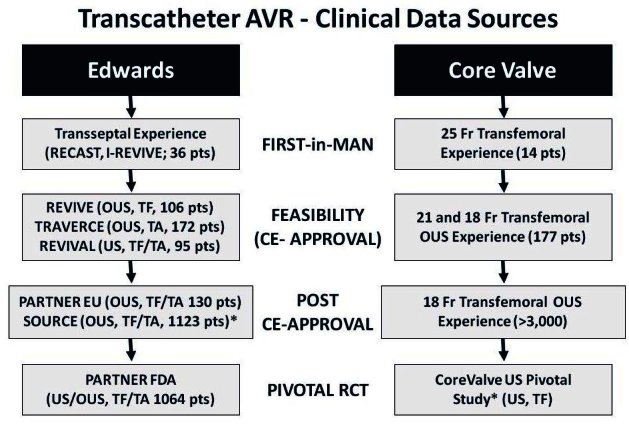
Transcatheter aortic valve implantation: clinical data sources.

Figure 5 summarizes current data on the use of Edwards and CoreValve percutaneous aortic prostheses. First-in-man small single-center registries documented the feasibility of the procedure using first-generation systems on a compassionate basis in high-risk patients who were denied surgical AVR. This was followed by larger multicenter registries that used further technical modifications aimed at refinement of procedural outcomes by reduction of the degree of perivalvular regurgitation, optimized valve positioning, and reduction of vascular complications.^15^–^23^ Based on the improved results from these registries, on May 16, 2007 the CoreValve company has received Communite European (CE) mark approval for its CoreValve ReValving^™^ System, and on September 5, 2007, Edwards Lifesciences received the CE mark for its Edwards SAPIEN THV for the treatment of high-risk patients with symptomatic AS.

## RANDOMIZED CONTROLLED TRIALS

**Figure 6:**
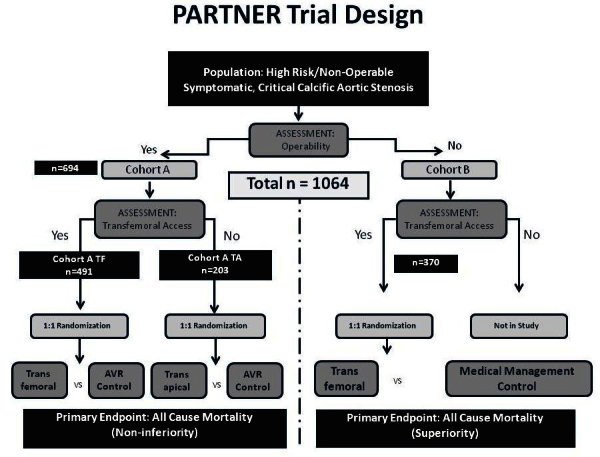
PARTNER trial design.

The Placement of AoRtic TraNscathetER Valves (PARTNER) trial is the pivotal investigational device exemption (IDE) randomized controlled comparison of patients with severe symptomatic AS randomly assigned to surgical AVR versus TAVI using Edwards SAPIEN THV implanted through either the femoral or apical approach (cohort A) or standard management (medical management with or without balloon angioplasty) versus TAVI (cohort B) if surgery is contraindicated (Figure 6).^24^ Primary outcome measure is 1-year survival. Secondary outcome measures include change in functional status from base-line per New York Heart Association (NYHA) classification, freedom from major adverse cardiac events, evidence of prosthetic valve dysfunction (hemolysis, infection, thrombosis, severe paravalvular leak, or migration), duration of hospitalization, quality of life, and rate of recurrent hospitalization. Careful screening of the patients was a crucial part of the trial. To be included the patients underwent scrutinized clinical evaluation, echocardiographic assessment, coronary angiography, and vascular access assessment including peripheral computer tomography and/or intravascular ultrasound. Every case was then reviewed by cardiac surgeons and interventional cardiologists for the cohort assignment and treatment strategy. As of today, enrollment of 1,064 patients has been completed, and the outcomes with TAVI as compared with standard therapy among the patients who were not suitable candidates for surgery (cohort B) have been announced at the annual Transcatheter Therapeutics meeting in Washington in September this year.^25^ At 1 year, all-cause mortality was 30.7% with TAVI, as compared with 50.7% with standard therapy (hazard ratio with TAVI, 0.55; 95% confidence interval [CI], 0.40 to 0.74; P<0.001). The rate of the composite end point of death from any cause or repeat hospitalization was 42.5% with TAVI as compared with 71.6% with standard therapy (hazard ratio, 0.46; 95% CI, 0.35 to 0.59; P<0.001). The rate of cardiac symptoms was also remarkably lower at 1 year in patients who underwent TAVI vs. those who received standard therapy (25.2% vs. 58%; P<o.001). 25

After the completion enrollment of the patients in the PARTNER Trial, the US Food and Drug Administration (FDA) granted investigational device exemption for the non-randomized continued access to the Edwards SAPIEN valve to actively enrolling PARTNER sites to collect further safety and effectiveness data on the device.

The CoreValve US pivotal randomized trial is currently pending FDA approval.

## PATIENT POPULATION

Patient populations in the published registries are characterized by high-risk features including high percentage of octogenarians, presence of serious co-morbidities, and previous cardiac surgery. Specifically, in the combined analysis of Registry of EndoVascular Implantation of Valves in Europe (I-REVIVE) and tRanscatheter EndoVascular Implantation of VALves II (REVIVAL) registry on 161 patients treated with Edwards SAPIEN THV, 75% of the population were 80 years of age or older, more than a quarter of the patients had previous cardiac surgery, and more than a fifth of the patients had chronic obstructive pulmonary disease, peripheral artery disease, chronic renal insufficiency, and/or diabetes.^26^ A history of major or minor stroke, chest radiation, and an extensively calcified (porcelain) aorta are also quite prevalent among the patients undergoing TAVI, all resulting in a high logistic EuroSCORE. Likewise, in the randomized PARTNER trial, there was a high prevalence of a porcelain aorta (15.1%), chest deformity or deleterious effects of chest-wall irradiation (13.1%), respiratory insufficiency (23.5%), and frailty (23.1%). 25

## HEMODYNAMIC OUTCOMES

**Figure 7:**
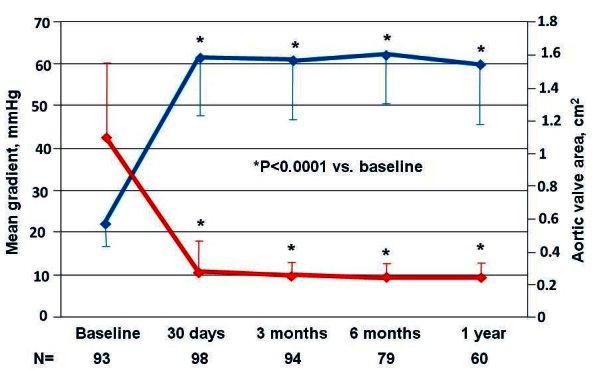
Transaortic valve gradient (red line) and aortic valve effective area (blue line) pre-procedure and at follow-up in the pooled analysis from the I-REVIVE and REVIVAL trials.

Once the aortic prosthesis is successfully implanted, the hemodynamic status improves immediately, including reduction in transvalvular gradient and increase in aortic valve effective orifice area (Figure 7). Post-procedural aortic regurgitation mainly due to paravalvular leak is a frequent finding but is trace to mild in the majority of cases. Pooled analysis from I-REVIVE and REVIVAL trials showed increased left ventricle ejection fraction in patients treated with TAVI and having available paired echocardiographic data and minimal follow-up of 30 days.^26^

## CLINICAL OUTCOMES

Early clinical outcomes are mostly impacted by two major factors including patient selection and operator experience. Thirty-day mortality has been diminishing from approximately 15% in early series to 7% in recent series.^15^–^23^ Improvement in techniques is an important contributor to the better clinical outcomes. In the multicenter European registry on 136 consecutive patients with severe symptomatic AS treated with CoreValve prosthesis, the overall 30-day mortality was 40%, 8.3%, and 10.8% for first-, second-, and third-generation of device, respectively, and the combined rate of death/stroke/myocardial infarction was 40.0%, 20.8%, and 14.7%, respectively.^22^

**Figure 8:**
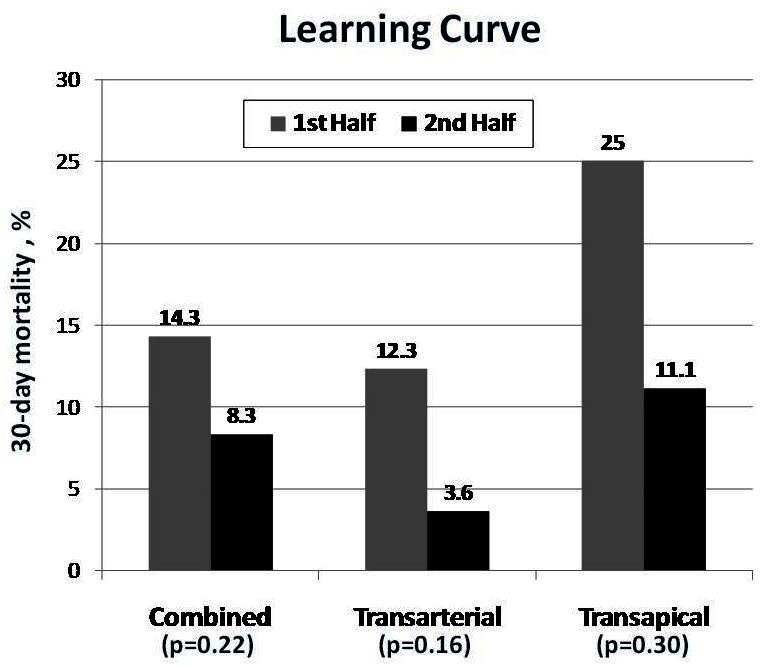
Thirty-day mortality as a function of learning curve: Vancouver experience. Figure included with permission from Circulation (Webb. Circulation 2009;119: 3009)^27^

**Figure 9:**
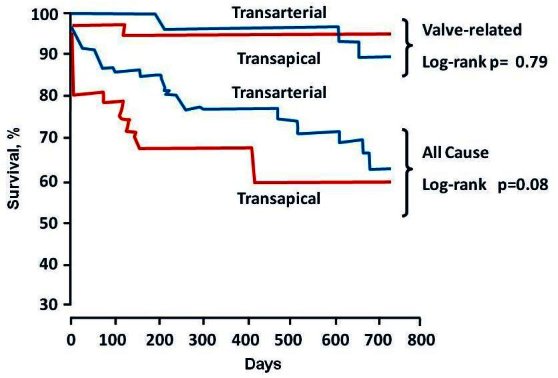
Kaplan-Meier estimates of all-cause mortality and valve-related mortality at 1 after transaortic valve replacement via transfemoral and transapical approach: Vancouver experience. Figure included with permission from Circulation (Webb. Circulation 2009;119:3009)^27^

Late clinical outcomes are largely determined by non-valve-related co-morbidities, concomitant severe mitral regurgitation, prior coronary artery by-pass grafting, and most series still demonstrate a 25%–30% 1-year mortality.^15^–^22^ An initial learning curve is obvious in the general experience (Figure 8). Mortality has been shown to be higher using transapical rather than transfemoral approach, which is probably related to higher patient risk profile (Figure 9).^20^,^27^

**Figure 10.**
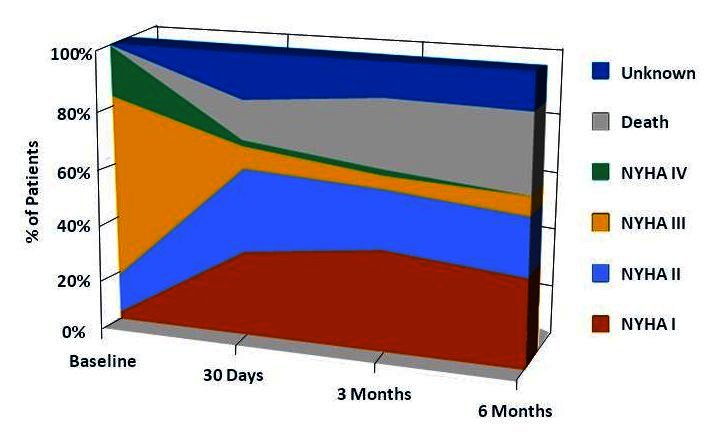
New York Heart Association functional class pre-procedure and at follow-up in the pooled analysis fromthe REVIVE, REVIVAL, TRAVERCE, and PARTNER EU trials.

TAVI survivors have undeniable and sustained clinical benefit as assessed by the NYHA functional capacity (Figure 10). In the multicenter European registry, overall functional status assessed by NYHA class improved from 3.3 ± 0.5 pre-procedure to 1.7 ± 0.7 post-valve implantation (*P* < 0.001). At 1-year follow-up, 90% of patients are in functional class I or II.^22^

## PROCEDURE-RELATED COMPLICATIONS

### VASCULAR INJURY

Major vascular injury, primarily iliofemoral dissection or perforation, is the most common TAVI complication performed through femoral approach. It occurred in 8% of 168 patients in the Vancouver series treated with the balloon-expandable Edwards SAPIEN percutaneous aortic valve.^27^ In the PARTNER trial, at 30 days, TAVI, as compared with standard therapy, was associated with a higher incidence of major vascular complications (16.2% vs. 1.1%, P<0.001) and major bleeding (16.8% vs. 3.9%, P<0.001).^25^ Major vascular complications are closely related to early mortality. In the pooled analysis from I-REVIVE and REVIVAL trials, occurrence of major vascular complications was associated with more than 3-fold increase in-hospital mortality (36% versus 10%).^26^,^28^ The development of lower-profile and less traumatic catheters, along with improved case selection and growing experience, results in remarkably reduced risk of vascular injury. For instance, in Columbia University Medical Center the incidence of major vascular complications decreased from 30% during the first 6 months of TAVI to 5.8% during the later period.

### NEUROLOGICAL EVENTS

Almost all patients (84%) undergoing TAVI have been demonstrated to have new foci of restricted diffusion on cerebral magnetic resonance imaging.^29^,^30^ Even multiple, these foci are typically not associated with apparent neurological events or measurable deterioration of neurocognitive function in up to 3 months of follow-up. However, risk of stroke is still high after TAVI, with a reported incidence of up to 9%.^29^ In the PARTNER trial, there was notably higher incidence of major strokes in patients treated with TAVI as opposed to standard treatment (5.0% vs. 1.1%, P = 0.06).^25^ The main sources of stroke in patients undergoing TAVI include embolic events from aorta, left ventricle, native or prosthetic valve, procedure-related aortic dissections, ischemia from hypotension during the procedure, or hemorrhagic complications associated with adjunctive pharmacotherapy. Approaches to reducing the risk include scrupulous pre-procedural screening for friable aortic atheroma, improvement in techniques to a gentle passage of catheters through the aortic arch, possible use of embolic protection devices, and optimization of anticoagulation strategies.

### ACUTE RENAL FAILURE

Post-procedural acute renal failure develops in approximately 12% of the patients treated with TAVI.^31^ Up to 2% of the patients may require temporary or permanent renal replacement therapy.^31^ By multivariate analysis, chronic renal failure is the most powerful independent predictor of mortality at late follow-up. Careful hemodynamic monitoring, maintenance of stable hemodynamics, the use of less traumatic catheters, and reduction in volume of contrast media are the key issues to reduce rates of procedure-related renal function deterioration.

### PROSTHETIC VALVE DYSFUNCTION

There is a higher frequency of paravalvular prosthetic aortic regurgitation associated with current TAVI devices^22^,^32^–^38^ compared to surgical AVR.^39^–^41^ The occurrence of significant aortic regurgitation (grade 2/4 or more) is typically related to prosthesis/annulus incongruence.^37^ To minimize paravalvular aortic regurgitation, appropriate annular measurements and prosthesis sizing are critical. To manage either paravalvular or central severe paravalvular prosthetic aortic regurgitation, repeat procedures including balloon angioplasty and repeat TAVI (valve-in-valve) may be required after the index TAVI.^42^,^43^

One matched-control retrospective study compared the hemodynamic performance of the aortic prosthesis performed using the Cribier-Edwards or Edwards SAPIEN bioprosthetic valve (*n* = 50) versus surgical aortic valve replacement using a stented valve (*n* = 50) or a stentless valve (*n* = 50).^38^ The groups were matched for gender, aortic annulus diameter, left ventricular ejection fraction, body surface area, and body mass index. Both at discharge and at 6- to 12-month follow-up, patients treated with TAVI compared to the patients treated with surgical bioprostheses had superior hemodynamic performance in terms of lower transprosthetic gradient but approximately 5-fold higher incidence of mild or moderate aortic regurgitation. There is an urgent need to develop strict definitions and to understand better the long-term clinical implications of paravalvular prosthetic aortic regurgitation. Up until now, the precise grading of paravalvular aortic regurgitation remains controversial.

### OTHER PROSTHESIS-RELATED ADVERSE EVENTS

Depending on the design characteristics and device position, prosthetic aortic valves may come in close contact with the anterior mitral valve leaflet, the intervalvular fibrosa, the aortic annulus, the ventricular septum, the aortic sinuses and root, the coronary arteries, and the cardiac conduction system. As such, prosthetic aortic valve procedures, and in particular TAVI, may have untoward effects on any of these structures that may result in important clinical consequences. New conduction disturbances develop in approximately 5% of the patients treated with Edwards THV and up to 40% of the patients treated with CoreValve ReValving^™^ system.^22^,^25^,^28^,^44^ The majority of these patients require permanent pace-maker. In one series of 34 patients treated with CoreValve ReValving^™^ system, pre-existing disturbance of cardiac conduction, a narrow left ventricular outflow tract, and the severity of mitral annular calcification predicted the need for permanent pacing.^45^ In the same series, the depth of prosthesis implantation was the only factor predictive for new-onset left bundle branch block.

Coronary artery obstruction, a serious complication of TAVI, occurs in less than 1% of the cases and frequently necessitates an emergency revascularization with percutaneous coronary intervention or coronary by-pass grafting.^27^,^46^ Possible mechanisms for coronary obstruction include impingement of the coronary ostia by the valve support structure in the setting of suboptimal valve positioning, embolization from calcium/ thrombus, or displacement of native aortic valve leaflets towards the coronary ostia during TAVI.20,27 Other relatively rare complications of TAVI include valve migration and valve embolization.

## VALVE ACADEMIC RESEARCH CONSORTIUM

The frequent changes in technology and procedure techniques, data collection processes, along with a lack of clearly determined and/or standardized study end-points have resulted in significant heterogeneity of the outcomes. In addition, the majority of studies lacked sufficient follow-up of essential valve-related end-points (e.g. valve performance as assessed by echocardiography). All this coupled with the complexity of patient population has created a “clinical data conundrum” and a critical need for a process to select and meticulously characterize clinical end-points in patients undergoing TAVI. With the recognition that consistency across end-point definitions is critical to the process of further data analysis and research, several academic research organizations from the United States and Europe, joined by representatives from the FDA and device manufacturers, combined their effort in a collaboration termed the Valve Academic Research Consortium (VARC). Two meetings, in San Francisco, California (September, 2009) and in Amsterdam, the Netherlands (December, 2009), including key physician experts, and representatives from the US Food and Drug Administration and device manufacturers, were focused on creating consistent end-point definitions and consensus recommendations for implementation in TAVI clinical research programs. The VARC consensus document is underway and will provide the combined expertise of cardiac surgeons, interventional cardiologists, general cardiologists, imaging specialists, clinical trialists, and other authorities in selecting the appropriate clinical end-points and their universal definitions that may be efficiently applied for regulatory and clinical trial purposes.^47^

## CONCLUSION AND FUTURE DIRECTIONS

A significant proportion of patients with severe AS do not undergo surgical AVR due to high-risk features. Transcatheter aortic valve implantation currently offers a definitive therapeutic solution to these patients. Clinical outcomes have stabilized in experienced hands, with a 30-day mortality of less than 10%. Careful patient selection, growing operator experience, and integrated multidisciplinary team approach contribute to notable improvement in outcomes. A step-up in functional status after successful TAVI is undeniable. The first randomized PARTNER trial convincingly demonstrated significant survival benefit along with improvement in cardiac symptoms in patients with severe AS not suitable candidates for surgical AVR treated with TAVI as compared to standard management. TAVI therefore should be the new standard of care for these patients. The results of randomized comparison of TAVI with AVR among high-risk patients with AS for whom surgery is a viable option (cohort A of the PARTNER trial) are eagerly awaited to provide further evidence on the applicability of TAVI in these patients. Data on long-term durability of the valves are lacking and should be carefully collected and analyzed. TAVI technology continues to evolve rapidly. Second-generation transcatheter aortic valve prototypes, such as the Lotus (Sadra Medical, Saratoga, California, USA), AorTx (Hansen Medical, Mountain View, California), Direct Flow (Direct Flow Medical, Inc., Santa Rosa, California), JenaValve (JenaValve Technology, Inc., Wilmington, Delaware, USA), The Heart Leaflet Technologies (HLT) valve (Heart Leaflet Technologies, Inc., Maple Grove, Minnesota, USA), Ventor Embracer™ (Ventor Technogies, Netanya, Israel), and Paniagua PHV (Endoluminal Technology Research, Miami, Florida, USA) are currently undergoing first-in-man implantations. Future clinical targets may include implantation of valve-in-valve for bioprosthetic aortic valve failure, TAVI in patients with lower risk AS, and treatment of patients with concomitant coronary artery disease and patients with low-flow low-gradient AS.

## Abbreviations:

AS: aortic stenosis;
AVR: aortic valve replacement;
CE: Communite European;
FDA: Food and Drug Administration;
IDE: investigational device exemption;
NYHA: New York Heart Association;
TAVI: transcatheter aortic valve implantation;
THV: transcatheter heart valve;
VARC: the Valve Academic Research Consortium.
